# A multi-class predictor based on a probabilistic model: application to gene expression profiling-based diagnosis of thyroid tumors

**DOI:** 10.1186/1471-2164-7-190

**Published:** 2006-07-27

**Authors:** Naoto Yukinawa, Shigeyuki Oba, Kikuya Kato, Kazuya Taniguchi, Kyoko Iwao-Koizumi, Yasuhiro Tamaki, Shinzaburo Noguchi, Shin Ishii

**Affiliations:** 1 Laboratory of Theoretical Life Science, Graduate School of Information Sciences, Nara Institute of Science and Technology, 8916-5 Takayama, Nara, 630-0101, Japan; 2 Osaka Medical Center for Cancer and Cardiovascular Diseases, 1-3-2, Nakamichi, Higashinari-ku, Osaka, 537-8511, Japan; 3 Department of Surgical Oncology, Osaka University Medical School, 2-2 Yamadaoka, Suita-ku, Osaka, 565-0871, Japan

## Abstract

**Background:**

Although microscopic diagnosis has been playing the decisive role in cancer diagnostics, there have been cases in which it does not satisfy the clinical need. Differential diagnosis of malignant and benign thyroid tissues is one such case, and supplementary diagnosis such as that by gene expression profile is expected.

**Results:**

With four thyroid tissue types, i.e., papillary carcinoma, follicular carcinoma, follicular adenoma, and normal thyroid, we performed gene expression profiling with adaptor-tagged competitive PCR, a high-throughput RT-PCR technique. For differential diagnosis, we applied a novel multi-class predictor, introducing probabilistic outputs. Multi-class predictors were constructed using various combinations of binary classifiers. The learning set included 119 samples, and the predictors were evaluated by strict leave-one-out cross validation. Trials included classical combinations, i.e., one-to-one, one-to-the-rest, but the predictor using more combination exhibited the better prediction accuracy. This characteristic was consistent with other gene expression data sets. The performance of the selected predictor was then tested with an independent set consisting of 49 samples. The resulting test prediction accuracy was 85.7%.

**Conclusion:**

Molecular diagnosis of thyroid tissues is feasible by gene expression profiling, and the current level is promising towards the automatic diagnostic tool to complement the present medical procedures. A multi-class predictor with an exhaustive combination of binary classifiers could achieve a higher prediction accuracy than those with classical combinations and other predictors such as multi-class SVM. The probabilistic outputs of the predictor offer more detailed information for each sample, which enables visualization of each sample in low-dimensional classification spaces. These new concepts should help to improve the multi-class classification including that of cancer tissues.

## Background

Histopathological analysis is the traditional mainstay of cancer diagnostics, and is relied on heavily to discriminate between malignant and benign tissues. However, in spite of its long and successful history as a routine medical technique, it is plagued by a number of well-known problems. For example, histological diagnosis often depends upon judgment calls by individual pathologists, leading to variations in diagnosis [1,2]. In addition, it may be difficult in some cases to differentiate between malignant and benign tissues. In such cases, microscopic observations have often been complemented by staining for molecular markers, such as expressed genes. This led directly to today's emerging approach, gene expression profiling, by which the expression levels of thousands of genes throughout the genome are measured by DNA microarrays or alternative techniques. Using this technique, diagnostic systems using multiple genes have been constructed [3]. Clinical status parameters such as prognosis or drug resistance are the popular targets, and the supervised learning theory is often applied.

In most examples of cancer classification, binary classifiers have been used, with multi-class predictors only rarely applied. However, because more than three tissue types are often differentiated during pathological diagnosis, a stable or optimized multi-class predictor is needed.

In this study, we developed a new multi-class predictor based on a probabilistic model. We examined various combinations of binary classifiers to perform optimized multi-class prediction, and applied our system to gene expression data from four tissue types of human thyroid origin. Thyroid cancer is a relatively common cancer, accounting for roughly 1% of total cancer incidence. There are two main types of thyroid cancer, papillary carcinoma (PC) and follicular carcinoma (FC). In addition to these malignant types, a benign tumor, follicular adenoma (FA), is also prevalent. The intriguing problem in thyroid cancer therapy is the preoperative differential diagnosis of follicular carcinoma vs. adenoma. The main diagnostic procedure is fine needle aspiration, but because the tissue structure is disrupted during the sampling process, the differential diagnosis is extremely difficult [4]. Papillary carcinoma is relatively easier to distinguish, but good molecular diagnostics would still be of potential clinical value. Thus, thyroid cancer represents a good example of a tumor type for which conventional histopathologic diagnosis faces limitations.

We performed gene expression profiling of the four thyroid tissues by adaptor-tagged competitive polymerase chain reaction (ATAC-PCR) [5,6], a high-throughput RT-PCR technique. We first analyzed the global differences in gene expression among the four thyroid tissues, and confirmed that the data matrix contained information for class separation. Then, we applied our new multi-class predictor for differential diagnosis of the four tissue types. The multi-class predictor outperformed previous methods used in the field.

## Results

### Difference in gene expression among the four thyroid tissues

**Figure 1:**
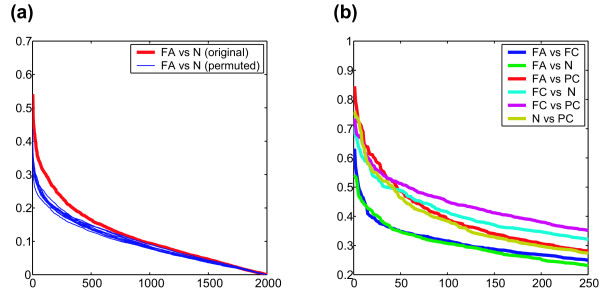
Correlation of global gene expression profiles with differences in thyroid tissue type. a) correlation ratio between follicular adenoma (FA) and normal thyroid (N). The red line and blue lines represent the correlation ratio of the original data and those of the permuted data, respectively. Blue lines are the results of twelve trials of permutation. b) correlation ratios of various combination of the four thyroid tissues.

We first examined whether there were significant differences in gene expression between the four thyroid tissue types by performing spectrum analysis based on correlation ratio (CR) [7]. Correlation ratio is an indicator for correlation of gene expression with two classes. High CR values indicate strong gene expression differences. Genes were first sorted by CR value order, and then the CRs of the original total data set were compared with those of permuted data. Figure 1a shows the results of comparison between FA and normal thyroid tissue (N). The CRs of the original data set are consistently higher than those of the permuted data. Because those of the original data were consistently higher throughout the full range of CRs, the correlation was not restricted to a small number of genes, but was a global character. With five other comparisons, we achieved similar results (data not shown), which suggested that there were significant differences in gene expression among the four thyroid tissues. The maximal (i.e. the most pessimistic) permutation p-values of the top 250 genes are 0.0064 for FA vs. FC, 0.0046 for FA vs. N, 0.0001 FC vs. N, and <10^-7 ^for the other four comparisons.

We then compared the CR spectra of all pairwise comparisons. The CR of each pair was calculated; genes were sorted by CR value order, and plotted (Figure 1b). Figure 1b shows the CR values of the top 250 genes: this figure represents the degree of gene expression difference of pairwise comparison, not indicating significance of gene expression difference (see below). Throughout this region, FA vs. N and FA vs. FC exhibited the smallest and the second smallest CR values. This result may reflect the difficulty in diagnosis: FA and N maintain microscopic follicular structures; FA and FC are the most difficult tissues to be differentially diagnosed. The other four comparisons, i.e., FC vs. N, FC vs. PC, FA vs. PC, N vs. PC, exhibited no substantial difference in CR in the high CR region (top 50 genes). In the middle CR region (the top 50–250 genes), the CRs of N vs. PC are close to those of FA vs. FC.

The CR spectra directly represent global differences in gene expression. We also evaluated these results by conventional measures, using p-values and q-values [8] of the standard t-test. When the cut-off p-value (significance level) is set at 0.0001, the numbers of genes and corresponding q-values are as follows: FA vs. N, 8 genes (q-value, 0.018); FA vs. FC, 7 (0.017); N vs. PC, 44 (0.0038); FC vs. N, 25 (0.0048); PC vs. FA, 66 (0.002); PC vs. FC, 41 (0.0026). These results parallel those from the CR spectrum analysis. Comparisons such as FA vs. N and FA vs. FC exhibited small numbers of significant genes and high q-values, and those such as PC vs. FA, and PC vs. FC exhibited large numbers of significant genes and low q-values. FC vs. N and N vs. PC are the middle of the above two.

### Differential diagnosis by binary classifier

**Figure 2:**
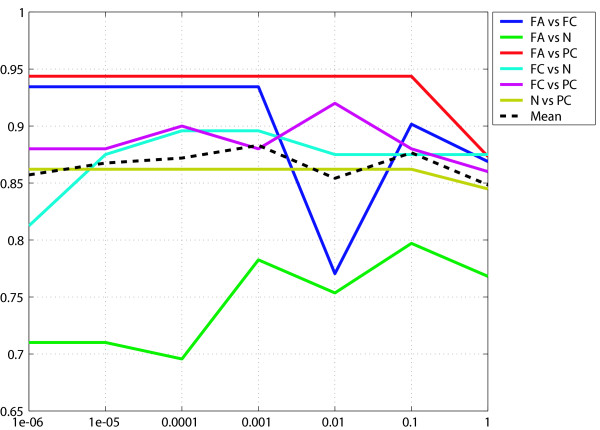
Accuracy curves of binary classifiers differentiating two of the four thyroid tissues. Vertical axis, accuracy; horizontal axis, selection criteria of diagnostic genes as p-value (t-statistics).

Before performing multi-class prediction, we examined the performance of binary classifiers in the differential diagnosis of each pair of the four thyroid tissues. We constructed each diagnostic system based on the weighted-voting algorithm [3], which has excellent predictive ability for gene expression data. As shown in Figure 2, classifiers for PC and one of the other tissues are stable, and exhibited good classification. In contrast, those for FA vs. FC and FA vs. N were not optimal, exhibiting unstable accuracy curves. Thus, the results using binary classifiers paralleled those of statistical analyses of individual genes by CR and false discovery ratio (FDR).

### Strategy for construction of a multi-class predictor

In this study, we constructed a multi-class prediction system based on a combination of binary classifiers. Each unit binary classifier was constructed by the weighted-voting algorithm, where the diagnostic genes were selected by the criterion *p *≤ 0.0001. In this criterion, all of the corresponding q-values were less than 0.05, maintaining the least inclusion of false positive genes. It should be noted that application of the following strategy is not limited to weighted-voting. We examined the following four types of construction methods using different numbers of binary classifiers.

*1R*: constructed using classifiers discriminating between one class and all of the other classes. *1R *is conventionally referred to as "one-to-the-rest" [9,10].

*11*: constructed using classifiers discriminating a pair of classes. *11 *is conventionally referred to as "one-to-one" [11-13].

*1A*: constructed using classifiers discriminating between one class and any subset of other classes.

*AA*: constructed using classifiers discriminating between any pair of disjoint subsets of classes.

**Figure 3:**
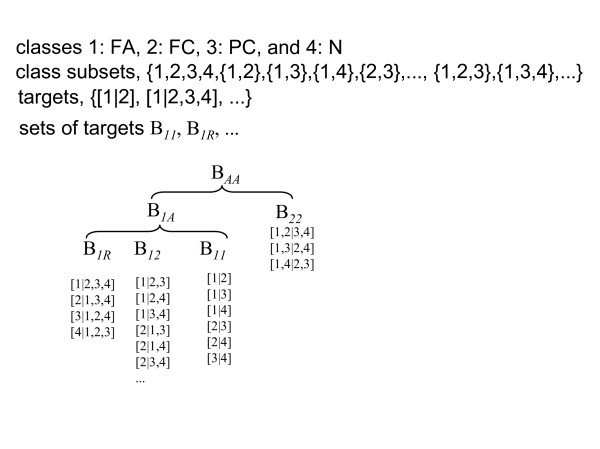
Combinations of unit binary classifiers used for construction of a multi-class predictor in this study. [*l*|*m*] represents a unit binary classifier separating class(es) *l *and class(es) *m*.

For this study of the four thyroid tissues, the numbers of classifiers were four (*1R*), six (*11*), twenty-two (*1A*), and twenty-five (*AA*). *1A *includes all of the binary classifiers of *1R *and *11*, and *AA *includes all of the binary classifiers of *1R*, *11 *and *1A*. The combinations in this study are shown in Figure 3. *1R *and *11 *have been used in previous studies [9-13], but *1A *and *AA *are novel.

The conventional construction rule for multi-class prediction from outputs of each binary classifier was designed for *11 *and *1R*. Binary output functions are usually used (see the materials and methods section), and prediction is performed by simple summation of the outputs. It should be noted that outputs are null when a group of classes, i.e., ''rest'' of ''one-to-the-rest'', wins. This rule, however, is not applicable to *AA*, because *AA *includes classification between groups of classes.

**Figure 4:**
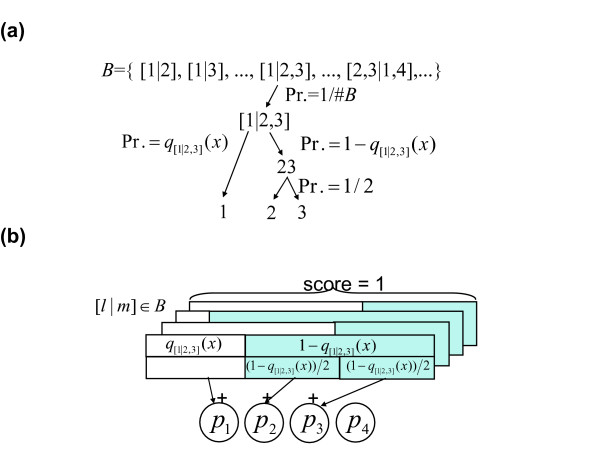
a) The probabilistic decision process of the multi-class predictor, for an example classification problem of four classes: 1, 2, 3 and 4. Firstly, one of the binary classifiers in a set *B *is selected with uniform probability 1#*B*, where #*B *is the number of binary targets in *B*. Secondly, class subset 1 or 2,3 is selected with probability *q*_[1|23]_(*x*) or 1 - *q*_[1|23]_(*x*), respectively. Thirdly, class 2 or 3 is selected with a probability of 1/2. Accordingly, one of the classes is selected with a certain probability. b) Calculation of class probability by *SHS*. For a binary classifier [*l*|*m*] in *B *and an input x which is a member of classes in *l *or *m*, we define *q*_[*l*|*m*]_(*x*) as an estimated probability where x is a member of class(es) in *l*, and the complement probability 1 - *q*_[*l*|*m*]_(*x*) where x is a member of class(es) in *m*. For example, *q*_[1|23]_(*x*) and 1 - *q*_[1|23]_(*x*) indicate the probability that *x *belongs to the class 1, and that *x *belongs to the class 2 or 3 provided that x belongs to the class 1, 2 or 3. In the *SHS *procedure, the probabilistic outputs by the multiple classifiers are shared and integrated by multiple classes, leading to the estimated class membership probabilities: *p*_1_,*p*_2_,*p*_3 _and *p*_4_. When *l *and/or *m *are set of multiple classes, the corresponding probabilistic outputs are shared equally to each of the members. For example, *q*_[1|23]_(*x*) is added to *p*_1_, 1 - *q*_[1|23]_(*x*) is shared equally and added to *p*_2 _and *p*_3_, *q*_[1|234]_(*x*) is added to *p*_1_, 1 - *q*_[1|234]_(*x*) is shared equally and added to *p*_2_, *p*_3 _and *p*_4_, and so on for all members of *B*. Consequently, we obtain an estimation of multiple class probabilities *p*_1_,*p*_2_, *p*_3 _and *p*_4 _by normalizing them so that the summation *p*_1_, *p*_2_, *p*_3 _and *p*_4 _would be one.

To incorporate general binary classifiers, we invented a method to integrate probabilistic outputs from the classifiers (Figure 4a). First, for each pair of class subsets, *l *or *m *(for example *l *= {*c*_1_} and *m *= {*c*_2_,*c*_3_}), the corresponding binary classifier was assumed to produce the probabilistic outputs *q*_[*l*|*m*] _and 1 - *q*_[*l*|*m*]_. When weighted voting is used as a classifier, the probabilistic output is calculated by one-dimensional logistic regression from the prediction strength of weighted voting. Thus, the probabilistic output (termed "class probability") of each of the multiple classes was calculated by integrating the set of probabilistic outputs calculated above. Finally, we obtained a single discrete class prediction as the class, which takes the maximum class probability. Using a simple integration method called simple summation (*SIS*), we calculated the sum of the probabilistic output *q*_[*l*|*m*] _to obtain the probability of each class, when the selected class subset *l *included the class. *SIS *is a probabilistic version of conventional voting [13,14]. In addition, we propose a method called shared summation (*SHS*), by which the probabilistic output of a multi-class group is shared equally by each class (Figure 4b). This method is based on a probabilistic decision process using probabilistic outputs from binary classifiers.

From four patterns of classifier combination (*1R, 11, 1A, AA*) and two output summation methods (*SIS *and *SHS*), we generated seven types of multi-class predictors (*AA *with *SIS *is not applicable).

### Classification of thyroid tissues

Using 119 samples from the learning set, we evaluated the seven multi-class predictors described above by leave-one-out cross-validation (LOO). For 119 iterations, one sample was left out, unit binary classifiers of an appropriate combination were constructed using data from the remaining 118 samples, each binary classifier was run, and the binary outputs were summated and classified. As shown in Table 1, prediction accuracies of *1A *and *AA *were higher than those of conventional methods, i.e., *11 *and *1R*. The prediction accuracy was greatest (79.8%) for *1A-SIS and 1A-SHS*, and the next for *AA-SHS *(79.0%).

**Figure 5:**
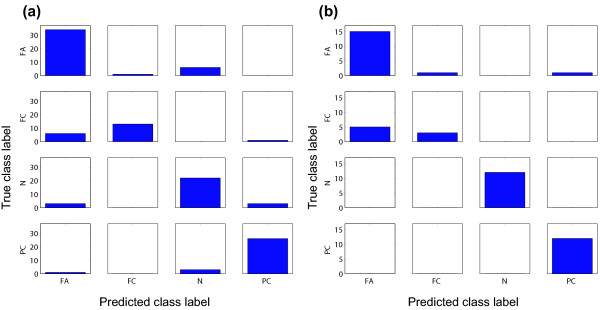
a) Confusion matrix of the results of the learning set (*1A-SHS*, evaluated by leave-one-out cross-validation). Each cell shows the number of samples along with true and predicted labels. True and predicted class labels are aligned vertically, and horizontally. b) Confusion matrix of the results of the test set.

The details of the predictor *1A*-*SHS *were analyzed by confusion matrix (Figure 5a). There were some misclassifications with FC as FA and FA as N. In contrast, there was very little misclassification of PC.

Our multi-class predictor was validated with an independent test set consisting of 49 samples. A multi-class predictor based on *1A-SIS, 1A-SHS*, and *AA*-*SHS *was constructed using gene expression data from the learning set (119 samples), and was applied to the independent test set. The overall prediction accuracy was 85.7% with all of the three predictors. The confusion matrix of *1A-SHS *is shown in Figure 5b.

### Comparison with other methods

We compared our method with other multi-class predictors popular in the field of cancer classification using the same learning data set (119 samples) as well as the other three data sets. The additional data sets are as follows. All data sets and algorithms were evaluated by LOO. With any algorithms, limited numbers of trials in classifier conditions (e.g., number of selected genes) were performed to avoid excessive overlearning.

#### The esophageal cancer data set

This data set is also composed of original gene expression profiles obtained from esophageal cancer of Japanese patients by ATAC-PCR [15]. The task here is differential diagnosis of three histological types: poorly differentiated, 14; moderately differentiated, 97; well differentiated, 30.

#### The GCM data set

The original global cancer map data set consists of 14 cancer types [14]. Because the number of combinations of binary classifiers increases exponentially as the number of classes increases, we selected five cancer types: breast, 11 samples; prostate, 10; leukemia, 30; renal, 11; mesothelioma, 11.

#### The SRBCT data set

Gene expression profiles about small round blue cell tumors (SRBCTs) of childhood, which contain 83 samples and 2308 genes measured by cDNA microarrays [16]. SRBCTs, which include the Ewing family of tumors (EWS), rhabdomyosarcoma (RMS), Burkitt lymphoma (BL), and neuroblastoma (NB) [17]. The composition of the samples are 29 (EWS), 25 (RMS), 11 (BL), and 18 (NB).

Firstly, we tested our predictors based on the weighted-voting algorithm with various diagnostic gene sets. We selected diagnostic genes with seven threshold p-values, i.e., 10^-6^, 10^-5^, 10^-4^, 10^-3^, 10^-2^, 10^-1^, 1. Due to unstable results with a small number of genes, we used at least 10 genes in cases where the number of selected genes was less than 10. In Table 2, we presented only best accuracies with respective threshold p-values. With the thyroid cancer data set, *1A-SHS/SIS *exhibited best results. *11-SHS *exhibited the accuracy as high as those by *1A-SHS/SIS*, but threshold p-value was their 10-fold. With the GCM set, *AA-SHS *exhibited the highest accuracy. With the esophageal cancer data set, the highest accuracy was observed with *1A/AA-SHS*. As a whole, these results suggest *1A *and *AA *are superior to conventional combinations of the classifiers, i.e., *11 *and *1R*.

Our predictors were compared with other multi-class prediction methods reported in the field. One is the combination of support vector machines (multi-class SVM, MC-SVM) described by Ramaswamy et al. [14], and the other is the shrunken centroid method (SC) [18]. The first method corresponds to *1R-SIS *without probabilistic outputs by our definition, and linear-kernel SVM is used as a unit binary classifier instead of a weighted-voting algorithm. To select diagnostic genes, we performed recursive feature elimination [19]: prediction accuracies were determined with various numbers of genes, i.e., all genes, 2000, 1024, 512, 256, 128, 64, 32, 16, 8, 4, 2, 1. The greatest accuracies with respective gene number are shown in Table 2: the accuracies were less than those obtained by our method.

The shrunken centroid is an improved method of the simple nearest prototype (centroid) classifier. We evaluated this method by LOO with various shrinkage parameters from 0 to 6 with 0.5 as an interval, i.e., a total of 13 parameters. With the all four data sets, our method yielded results superior to the shrunken centroid.

### Tissue sample display by class probability

**Figure 6:**
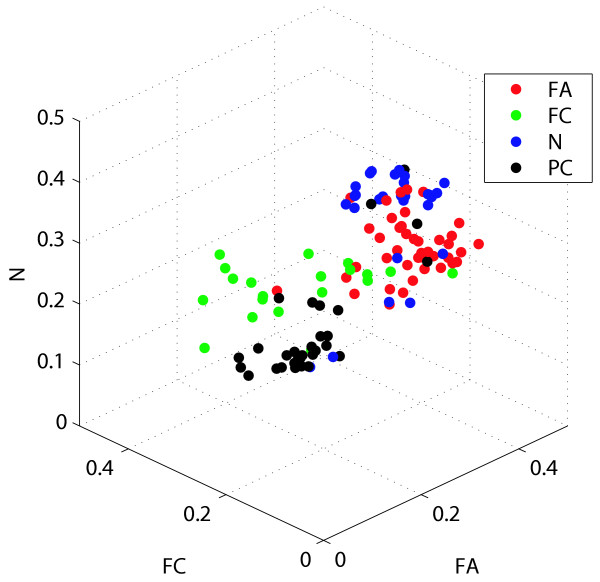
Visualization of the learning samples by class probabilities.

The outputs of our multi-class predictors include not only the label, but also the class probability of each class. This information can be used to differentiate samples under the same label, and further characterize differences among them. In Figure 6, the learning set samples are displayed in the three-dimensional space constructed from the class probabilities of FA, FC, and N, and indeed all four tissue types could be resolved into separate locations. It should be noted that several samples are located apart from their respective clusters, partly due to an error in labeling in the histopathological diagnosis. Otherwise, these could represent samples with molecular features distinct from the other members of their assigned clusters.

## Discussion

In spite of their integral role in cancer diagnosis, many histopathological diagnostic protocols are still dependent on judgment calls made by individual pathologists, and as a result are significantly prone to error. More objective measures are needed, such as computational interpretation of microscopic images or complementary diagnosis by gene expression. For diagnosis by gene expression profile, conventional binary classifiers are not sufficient because the number of tissue types to be differentially diagnosed is usually greater than two. In this study, we have described the differential diagnosis of four thyroid tissues by use of a novel multi-class predictor with a test prediction accuracy of as high as 79~85%. As a result, our diagnostic system represents a promising first step towards an automated pathological tool to complement current diagnostic procedures.

Our approach to multi-class prediction is a novel one characterized by increasing combination of binary classifiers and a probabilistic approach for outputs. Traditionally, "one-to-one" or "one-to-the-rest" approaches have been applied to integrate combinations of unit binary classifiers. As shown above, *1A *and *AA *exhibited prediction accuracy better than conventional approaches. Our results suggest that conventional methods such as "one-to-one" and "one-to-the-rest" are not necessarily optimal, recommending use of more combinations of binary classifiers. The main disadvantage of *AA *and *1A *is that the number of classifiers increases exponentially as the number of classes increases. However, this is not a significant disadvantage when the method is applied to cancer classification problems, because the number of classes for separation is nearly always less than seven.

Another characteristic of our predictor is the introduction of a probabilistic model to the outputs. Probabilistic output of the multi-class predictor, here designated as class probability, offers detailed information about each sample, which could not be obtained by binary outputs. In particular, it is useful to visualize the relationships between the classes using a two- or three-dimensional plot of class probabilities. This visualization may in fact be more effective than unsupervised approaches such as principal component analysis and multi-dimensional scaling [20], because the class probabilities were calculated from weighted sums of selected diagnostic genes. In particular, it is useful for gaining a deeper understanding of individual samples. For example, among the FC samples, some appear rather similar to FA, and a few seem separate from all of the classes. Detailed analysis of class probability may lead to discoveries of new tumor classes. It is also possible that some of the samples may have been initially misdiagnosed by the pathologist. Reexamination of these tissue sections may lead to more accurate diagnosis.

One may argue that our assumption that the choice of unit binary classifier is an independent process (see Materials and Methods) is too simplified. For example, because one class appears in different classifiers in different combinations, these classifiers may not be independent. A simple solution for this problem is introduction of prior probability in the choice of unit binary classifier. For example, our predictor is only modestly effective at separating FC from FA, but this shortcoming could be improved by the introduction of appropriate prior probabilities.

Another possible critique is our equal sharing of outputs of unit binary classifiers. The optimal ratio of output sharing can be obtained by taking account of correlations between similar targets, such as [*c*_1_|*c*_2_] and [*c*_1_,*c*_2_|*c*_3_,*c*_4_]. Our preliminary results using a method similar to that previously used by Hastie et al. in the pairwise case [12] suggested that the prediction accuracy was equal to that of *SHS*. We suspect that *SHS *is a good estimate for the sharing method deduced by this more detailed model. In any case, the optimum model of outputs should not decrease accuracy as increase in number of constituent binary classifiers. However, with the thyroid data set, the accuracy of *1A *was higher than that of *AA*, suggesting that the current probabilistic model is incomplete. Our complete version of the output model, requiring a larger computer resource, will be described elsewhere (Yukinawa et al., submitted).

Multi-class classification techniques can be roughly divided into two types. One is the decomposition of multi-class problems into binary ones. ''one-to-the-rest'' methods [9,10], pairwise comparisons, i.e., ''one-to-one'' methods [11-13], error-correcting output coding [21-23] belong to this type. There have been comprehensive studies centered on SVM [24,25], but there have been no definitive methods in this type. Recent developments also include logitBoost [26] or genetic algorithm [27] as unit classifiers. The other type is binary classification algorithms that can be naturally extended to handle multi-class problems directly. SVM with multiclass objective functions [28,29], discriminant analysis [30] and regression and decision trees are of this type. Recent developments include shrunken centroid [18], an advanced version of nearest centroid [31], and total principal component regression [32]. Our method is an extension of one of the former type approaches. For comparison with previous methods, we chose a ''one-to-the-rest'' method using a support vector machine from the first type [14], and the shrunken centroid method [18] from the latter type. With the four data sets, including two in the public domain, we established that our method was superior to these two methods.

Ramaswamy et al. [14] pointed out that the weighted-voting was generally not well-performed as MC-SVM. This may be because they selected genes in the order of signal-to-noise ratio, and used the gene number as the threshold. In contrast, we used the p-value as the threshold: the p-value threshold would be essential to obtain multi-class predictor with the weighed-voting algorithm, because using the number of genes instead may incorporate unnecessary genes or ignore necessary genes in some classifiers.

The shrunken centroid [18] is a simple modification of the nearest centroid method. The prediction accuracy obtained was higher than that of the combination of SVM, but still fell significantly short of our algorithm. Moreover, because the best results were obtained by comparison of different shrinkage parameters, the predictor may be overfitted. The discriminant hyperplanes resulting from the shrunken centroid were rather simple, and a more complex classification such as that for thyroid tissue may require more complicated discriminant surfaces, such those obtained by our method. One of the advantages of the shrunken centroid method is that each sample can be described by class probabilities calculated from discriminant scores. This feature is similar to our class probability, and enables visualization of samples as in our method.

The main shortcoming common to previous multi-class studies of cancer classification is the use of sample data matrices, which are easily diagnosed by conventional techniques. The solid tumors or small round blue cell tumors used have been of different tissues of origin, and could be differentiated easily by microscopic observations as well as by gene expression analysis. Because solving such problems is of little clinical value, such data matrices are not adequate for the benchmark test of predictors. Use of data matrices which model realistic problems is of pivotal importance for evaluation of techniques.

## Conclusion

Among tasks in histopathology, differential diagnosis of thyroid tissues is a difficult one. We have developed a new multi-class predictor based on a probabilistic model using gene expression data. Tests on four types of thyroid tissues revealed its excellent performance in prediction. The novel approaches introduced in this study show promise as a means to differentiate between similar tumor types from the same tissue of origin. However, before we can draw a conclusion on their efficacy, a number of confirmatory experiments, including analysis of artificial data sets, are necessary. Nevertheless, we believe these algorithms will contribute to significant advances in the pathological diagnosis of cancer in the near future.

## Methods

### Patients and tumor samples

The learning set consisted of 41 FA, 20 FC, 30 PC and 28 N samples, and the test set consisted of 17 FA, 8 FC, 12 PC and 12 N samples. For the test set, we simply chose samples with the most recent surgery dates. The tumor samples were obtained from patients who underwent surgery (hemithyroidectomy or total thyroidectomy) during the period from February 1998 to December 2002. Histological diagnosis was performed by an experienced pathologist. Tumor samples obtained during surgery were snap frozen in liquid nitrogen and stored at -80°C until use. The study protocol was approved by the Institutional Review Board of Osaka University Medical School, and written informed consent was obtained from each patient.

### ATAC-PCR assay and data processing

To select genes for ATAC-PCR, we first constructed four cDNA libraries; one from a mixture of five normal thyroid tissues, one from a mixture of five PC samples, one from a mixture of five FA samples and one from a mixture of four FC samples as described [33]. We then performed single pass sequencing of 6154 clones (1424 from follicular carcinoma, 1592 from papillary carcinoma, 1575 from follicular adenoma, and 1563 from normal thyroid). A total of 2383 genes were selected from this EST collection, prioritizing abundant genes. Then, 133 genes were added by experts in thyroid cancer, based on literature information. We designed PCR primers for ATAC-PCR reactions for these 2516 genes. The specificity of this gene selection provides an advantage over more universal gene sets, such as those selected from the UniGene database, which include genes not expressed in thyroid tissue. Total RNA was purified from thyroid tumors using Trizol reagent (GibcoBRL), and was subjected to gene-expression profiling by ATAC-PCR. The ATAC-PCR experimental procedure was performed as previously described [34]. The measurements of 2516 genes were repeated twice with ten tumor samples, and correlation coefficient of these duplicates was calculated. The correlation coefficient and corresponding p-value was 0.92 and 0.000163, showing the sufficient reproducibility of the measurement. The raw data describing gene expression levels were divided by the median of each sample. Because the median of each sample is expected to reflect the overall mRNA level, normalization by this value corrects for variation in mRNA level from sample to sample. Values less than 0.05 and more than 20 were converted to 0.05 and 20, respectively, and subsequently the entire data matrix was converted to a logarithmic scale. The detailed protocols for the ATAC-PCR experimental procedure are available on our web site [35].

For the analysis, missing values were estimated and filled in by a method based on Bayesian inference [36] (see the additional files 1 and 2). In the ATAC-PCR reaction, the fluorescence intensity of the products is correlated with the data quality. Genes were sorted by the order of the average of the fluorescence intensity, and we selected the top 2000 genes for the following analysis.

### Statistical analysis

The correlation ratio *R *of gene *i *is defined by the following equation.

R=∑c∈Cnc(x¯ci−x¯i)2∑i∑j(xij−x¯i)2
 MathType@MTEF@5@5@+=feaafiart1ev1aaatCvAUfKttLearuWrP9MDH5MBPbIqV92AaeXatLxBI9gBaebbnrfifHhDYfgasaacH8akY=wiFfYdH8Gipec8Eeeu0xXdbba9frFj0=OqFfea0dXdd9vqai=hGuQ8kuc9pgc9s8qqaq=dirpe0xb9q8qiLsFr0=vr0=vr0dc8meaabaqaciaacaGaaeqabaqabeGadaaakeaacqWGsbGucqGH9aqpdaGcaaqaamaalaaabaWaaabuaeaacqWGUbGBdaWgaaWcbaGaem4yamgabeaakiabcIcaOiqbdIha4zaaraWaaSbaaSqaaiabdogaJjabdMgaPbqabaGccqGHsislcuWG4baEgaqeamaaBaaaleaacqWGPbqAaeqaaOGaeiykaKYaaWbaaSqabeaacqaIYaGmaaaabaGaem4yamMaeyicI4Saem4qameabeqdcqGHris5aaGcbaWaaabuaeaadaaeqbqaaiabcIcaOiabdIha4naaBaaaleaacqWGPbqAcqWGQbGAaeqaaOGaeyOeI0IafmiEaGNbaebadaWgaaWcbaGaemyAaKgabeaakiabcMcaPmaaCaaaleqabaGaeGOmaidaaaqaaiabdQgaQbqab0GaeyyeIuoaaSqaaiabdMgaPbqab0GaeyyeIuoaaaaaleqaaaaa@5583@

where *n*_*c *_is the number of samples in a particular class *c *∈ *C*; *x*_*ij *_is the expression level of gene *i *in sample *j*; x¯
 MathType@MTEF@5@5@+=feaafiart1ev1aaatCvAUfKttLearuWrP9MDH5MBPbIqV92AaeXatLxBI9gBaebbnrfifHhDYfgasaacH8akY=wiFfYdH8Gipec8Eeeu0xXdbba9frFj0=OqFfea0dXdd9vqai=hGuQ8kuc9pgc9s8qqaq=dirpe0xb9q8qiLsFr0=vr0=vr0dc8meaabaqaciaacaGaaeqabaqabeGadaaakeaacuWG4baEgaqeaaaa@2E3D@_*ci *_is the average expression level of gene *i *for samples in a particular class *c *∈ *C*; and x¯
 MathType@MTEF@5@5@+=feaafiart1ev1aaatCvAUfKttLearuWrP9MDH5MBPbIqV92AaeXatLxBI9gBaebbnrfifHhDYfgasaacH8akY=wiFfYdH8Gipec8Eeeu0xXdbba9frFj0=OqFfea0dXdd9vqai=hGuQ8kuc9pgc9s8qqaq=dirpe0xb9q8qiLsFr0=vr0=vr0dc8meaabaqaciaacaGaaeqabaqabeGadaaakeaacuWG4baEgaqeaaaa@2E3D@_*i *_is the average expression level of gene *i *for all samples. Permutation p-value of a correlation ratio is defined by the proportion of trials where *R *of randomly permutated data exceeded that of original data, among 10,000 permutation trials.

p-values were calculated using t-statistics. q-values were calculated using software by Dr Storey [37].

### Unit binary classifiers

The data set used in this study is defined as *X *= {*x*^(*n*)^,*t*^(*n*)^}_*n *= 1:*N*_; here, *x*^(*n*) ^∈ ℜ^*D *^and *t*^(*n*) ^∈ *C *≡ {*c*_1_,...,*c*_*M*_} denote gene expression pattern and class label, respectively, of the *n*-th sample; *D *is the number of genes.

*C *is the set of whole classes, and 2^*c *^is the labels' power set, as follows.

2^*c *^≡ {{*c*_1_}, {*c*_2_},..., {*c*_1_, *c*_2_},..., {*c*_1_, *c*_2_, *c*_3_},..., *C*, *φ*}.

We designated a pair of subsets to be classified as a "target". Consider a binary classifier trained on a binary target [*l*|*m*] ∈ *B *where indices *l*, *m *∈ 2^*C *^- {*C*, *φ*} denote two disjoint (*l *∩ *m *= *φ*) subsets of class labels and *B *is a certain set of targets. In this study, we used some selections of *B *such as *B*_1*R*_, *B*_11_, *B*_1*A *_and *B*_*AA *_(Figure 3).

We employed the weighted-voting algorithm for the binary classification of each target. [*l*|*m*]. The discriminant function *f*_[*l*|*m*] _(*x*) ∈ ℜ was defined using selected diagnostic genes with the criterion *p *≤ 0.0001 for each gene-wise t-test.

### Output function of the binary classifier

When a discriminant function *f*_[*l*|*m*] _(*x*) is determined from a learning data set *X*_[*l*|*m*] _= {(*x*^(*n*)^, *t*^(*n*)^) ∈ *X*|*t*^(*n*) ^∈ *l *∪ *m*}, the binary output function of the classifier is described as follows.

q[l|m](x)={1if f[l|m](x)≥00if f[l|m](x)<0.
 MathType@MTEF@5@5@+=feaafiart1ev1aaatCvAUfKttLearuWrP9MDH5MBPbIqV92AaeXatLxBI9gBaebbnrfifHhDYfgasaacH8akY=wiFfYdH8Gipec8Eeeu0xXdbba9frFj0=OqFfea0dXdd9vqai=hGuQ8kuc9pgc9s8qqaq=dirpe0xb9q8qiLsFr0=vr0=vr0dc8meaabaqaciaacaGaaeqabaqabeGadaaakeaacqWGXbqCdaWgaaWcbaGaei4waSLaemiBaWMaeiiFaWNaemyBa0Maeiyxa0fabeaakiabcIcaOiabdIha4jabcMcaPiabg2da9maaceqabaqbaeqabiGaaaqaaiabigdaXaqaaiabbMgaPjabbAgaMjabbccaGiabdAgaMnaaBaaaleaacqGGBbWwcqWGSbaBcqGG8baFcqWGTbqBcqGGDbqxaeqaaOGaeiikaGIaemiEaGNaeiykaKIaeyyzImRaeGimaadabaGaeGimaadabaGaeeyAaKMaeeOzayMaeeiiaaIaemOzay2aaSbaaSqaaiabcUfaBjabdYgaSjabcYha8jabd2gaTjabc2faDbqabaGccqGGOaakcqWG4baEcqGGPaqkcqGH8aapcqaIWaamcqGGUaGlaaaacaGL7baaaaa@5FAF@

In this study, we took a probabilistic approach, where *q*_[*l*|*m*]_(·) may be a real value of 0 ≤ *q*_[*l*|*m*]_(·) ≤ 1 which corresponds to a posterior probability, Pr(*t *∈ *l*|*x*, *t *∈ *l *∪ *m*, *X*_[*l*|*m*]_). The following logistic function *r*(*x*; *a*_[*l*|*m*]_, *b*_[*l*|*m*]_) can represent a mapping from the discriminant function *f*_[*l*|*m*]_(*x*) to the probability. The mapping is based on one-dimensional logistic regression,

r(x;a[l|m],b[l|m])=11+exp⁡(−a[l|m]f[l|m](x)−b[l|m],
 MathType@MTEF@5@5@+=feaafiart1ev1aaatCvAUfKttLearuWrP9MDH5MBPbIqV92AaeXatLxBI9gBaebbnrfifHhDYfgasaacH8akY=wiFfYdH8Gipec8Eeeu0xXdbba9frFj0=OqFfea0dXdd9vqai=hGuQ8kuc9pgc9s8qqaq=dirpe0xb9q8qiLsFr0=vr0=vr0dc8meaabaqaciaacaGaaeqabaqabeGadaaakeaacqWGYbGCcqGGOaakcqWG4baEcqGG7aWocqWGHbqydaWgaaWcbaGaei4waSLaemiBaWMaeiiFaWNaemyBa0Maeiyxa0fabeaakiabcYcaSiabdkgaInaaBaaaleaacqGGBbWwcqWGSbaBcqGG8baFcqWGTbqBcqGGDbqxaeqaaOGaeiykaKIaeyypa0ZaaSaaaeaacqaIXaqmaeaacqaIXaqmcqGHRaWkcyGGLbqzcqGG4baEcqGGWbaCcqGGOaakcqGHsislcqWGHbqydaWgaaWcbaGaei4waSLaemiBaWMaeiiFaWNaemyBa0Maeiyxa0fabeaakiabdAgaMnaaBaaaleaacqGGBbWwcqWGSbaBcqGG8baFcqWGTbqBcqGGDbqxaeqaaOGaeiikaGIaemiEaGNaeiykaKIaeyOeI0IaemOyai2aaSbaaSqaaiabcUfaBjabdYgaSjabcYha8jabd2gaTjabc2faDbqabaaaaOGaeiilaWcaaa@6B52@

where *a*_[*l*|*m*] _and *b*_[*l*|*m*] _are determined by maximum-likelihood estimation so that *r*(*x*^(*n*)^; *a*_[*l*|*m*]_, *b*_[*l*|*m*]_) for all learning data *x*^(*n*) ^∈ *X*[_[*l*|*m*] _best fits the corresponding labels *t*^(*n*)^. The probabilistic output for the gene expression vector of a test sample *x*^NEW^,*q*_[*l*|*m*]_(*x*^NEW^), is determined as follows.

*q*_[*l*|*m*]_(*x*^NEW^) = *r*(*x*^NEW^; *a*_[*l*|*m*]_, *b*_[*l*|*m*]_).

### Multi-class prediction scheme

For each expression vector *x*, we considered a probabilistic process of selecting a single class *c *∈ *C *as a prediction of its label.

1. Random selection of a binary target [*l*|*m*] in a set *B*, and calculation of *q*_[*l*|*m*]_(*x*).

2. Stochastic selection of a class subset *l *or *m*, with probability *q*_[*l*|*m*]_(*x*) for *l *and 1 - *q*_[*l*|*m*]_(*x*) for *m*.

3. Random selection of a single class prediction *c *in the above selected subset *l *or *m*. According to this process, the probability of selecting a class *c *∈ *C*, referred to as "class probability", becomes

pc(x)=1Z∑[l|m]∈B{I(c∈l)q[l|m](x)/#l+I(c∈m)(1−q[l|m](x))/#m}.
 MathType@MTEF@5@5@+=feaafiart1ev1aaatCvAUfKttLearuWrP9MDH5MBPbIqV92AaeXatLxBI9gBaebbnrfifHhDYfgasaacH8akY=wiFfYdH8Gipec8Eeeu0xXdbba9frFj0=OqFfea0dXdd9vqai=hGuQ8kuc9pgc9s8qqaq=dirpe0xb9q8qiLsFr0=vr0=vr0dc8meaabaqaciaacaGaaeqabaqabeGadaaakeaacqWGWbaCdaWgaaWcbaGaem4yamgabeaakiabcIcaOiabdIha4jabcMcaPiabg2da9maalaaabaGaeGymaedabaGaemOwaOfaamaaqababaWaaiWabeaacqWGjbqscqGGOaakcqWGJbWycqGHiiIZcqWGSbaBcqGGPaqkcqWGXbqCdaWgaaWcbaGaei4waSLaemiBaWMaeiiFaWNaemyBa0Maeiyxa0fabeaakiabcIcaOiabdIha4jabcMcaPiabc+caViabcocaJiabdYgaSjabgUcaRiabdMeajjabcIcaOiabdogaJjabgIGiolabd2gaTjabcMcaPiabcIcaOiabigdaXiabgkHiTiabdghaXnaaBaaaleaacqGGBbWwcqWGSbaBcqGG8baFcqWGTbqBcqGGDbqxaeqaaOGaeiikaGIaemiEaGNaeiykaKIaeiykaKIaei4la8Iaei4iamIaemyBa0gacaGL7bGaayzFaaaaleaacqGGBbWwcqWGSbaBcqGG8baFcqWGTbqBcqGGDbqxcqGHiiIZcqWGcbGqaeqaniabggHiLdGccqGGUaGlaaa@7421@

Here *I*(*A*) is equal to 1 if condition *A *holds, and zero otherwise; #*l *and #*m *denote the numbers of classes in the class subset *l *and *m*; *Z *is a normalization constant which guarantees ∑c∈Cpc(x)=1
 MathType@MTEF@5@5@+=feaafiart1ev1aaatCvAUfKttLearuWrP9MDH5MBPbIqV92AaeXatLxBI9gBaebbnrfifHhDYfgasaacH8akY=wiFfYdH8Gipec8Eeeu0xXdbba9frFj0=OqFfea0dXdd9vqai=hGuQ8kuc9pgc9s8qqaq=dirpe0xb9q8qiLsFr0=vr0=vr0dc8meaabaqaciaacaGaaeqabaqabeGadaaakeaadaaeqbqaaiabdchaWnaaBaaaleaacqWGJbWyaeqaaOGaeiikaGIaemiEaGNaeiykaKIaeyypa0JaeGymaedaleaacqWGJbWycqGHiiIZcqWGdbWqaeqaniabggHiLdaaaa@3AC0@. The above equation takes the summation of *q*_[*l*|*m*]_(*x*) for all targets [*l*|*m*] where subset *l *or *m *contains class *c*, and where probability scores are shared equally to member classes of the subsets. Therefore, this is called shared summation (*SHS*) of probabilistic output. Subsequently, we can obtain a Bayes-optimal class prediction as arg⁡max⁡c∈Cpc(x)
 MathType@MTEF@5@5@+=feaafiart1ev1aaatCvAUfKttLearuWrP9MDH5MBPbIqV92AaeXatLxBI9gBaebbnrfifHhDYfgasaacH8akY=wiFfYdH8Gipec8Eeeu0xXdbba9frFj0=OqFfea0dXdd9vqai=hGuQ8kuc9pgc9s8qqaq=dirpe0xb9q8qiLsFr0=vr0=vr0dc8meaabaqaciaacaGaaeqabaqabeGadaaakeaadaWfqaqaaiGbcggaHjabckhaYjabcEgaNjGbc2gaTjabcggaHjabcIha4bWcbaGaem4yamMaeyicI4Saem4qameabeaakiabdchaWnaaBaaaleaacqWGJbWyaeqaaOGaeiikaGIaemiEaGNaeiykaKcaaa@3F1E@.

In simple summation (*SIS*), we determined class probability *p*_*c*_(*x*) for each class *c *∈ *C *as a summation of *q*_[*l*|*m*]_(*x*) for all targets [*l*|*m*] such that *l *or *m *is {*c*} itself,

pc(x)=1Z∑[l|m]∈B{I(l={c})q[l|m](x)+I(m={c})(1−q[l|m](x))},
 MathType@MTEF@5@5@+=feaafiart1ev1aaatCvAUfKttLearuWrP9MDH5MBPbIqV92AaeXatLxBI9gBaebbnrfifHhDYfgasaacH8akY=wiFfYdH8Gipec8Eeeu0xXdbba9frFj0=OqFfea0dXdd9vqai=hGuQ8kuc9pgc9s8qqaq=dirpe0xb9q8qiLsFr0=vr0=vr0dc8meaabaqaciaacaGaaeqabaqabeGadaaakeaacqWGWbaCdaWgaaWcbaGaem4yamgabeaakiabcIcaOiabdIha4jabcMcaPiabg2da9maalaaabaGaeGymaedabaGaemOwaOfaamaaqababaWaaiWabeaacqWGjbqscqGGOaakcqWGSbaBcqGH9aqpcqGG7bWEcqWGJbWycqGG9bqFcqGGPaqkcqWGXbqCdaWgaaWcbaGaei4waSLaemiBaWMaeiiFaWNaemyBa0Maeiyxa0fabeaakiabcIcaOiabdIha4jabcMcaPiabgUcaRiabdMeajjabcIcaOiabd2gaTjabg2da9iabcUha7jabdogaJjabc2ha9jabcMcaPiabcIcaOiabigdaXiabgkHiTiabdghaXnaaBaaaleaacqGGBbWwcqWGSbaBcqGG8baFcqWGTbqBcqGGDbqxaeqaaOGaeiikaGIaemiEaGNaeiykaKIaeiykaKcacaGL7bGaayzFaaaaleaacqGGBbWwcqWGSbaBcqGG8baFcqWGTbqBcqGGDbqxcqGHiiIZcqWGcbGqaeqaniabggHiLdGccqGGSaalaaa@72F5@

where *Z *is a normalization constant. Because of the condition *l *= {*c*} or *m *= {*c*}, classifiers whose output has more than one label were not included in the above summation. As a result, *AA *and *1A *are equivalent in the *SIS *case. Although *SIS *is a straightforward extension of conventional voting criterion to handle probabilistic guesses, it lacks a consistent model of decision process, such as that presents in the background of *SHS*.

Matlab programs to perform *1A/AA-SIS/SHS *using the weighted-voting algorithm is available from NY.

## Authors' contributions

NY and SO implemented their algorithm, and NY carried out application experiments to gene expression data sets. SO and SI developed the mathematical formulation of the multi-class classifiers proposed in this paper. KK initiated and supervised the whole project. KT and KI-K did experimental parts of the thyroid cancer study. YT and SN collected thyroid tumor and normal tissues, and are responsible for clinical aspects of the study. All authors read and approved the final manuscript.

## Supplementary Material

Additional File 1 contains all gene expression data. Click here for file

Additional File 2 contains explanation of the data file 1. Click here for file

## Figures and Tables

**Table 1:** Prediction accuracies (%) of the learning sample set, evaluated by leave-one-out cross-validation.

	*SIS*	*SHS*
1R	69.8	69.8
*11*	77.3	77.3
*1A*	79.8	79.8
*AA*	N/A	79.0

**Table 2:** Comparison of prediction accuracies of various algorithms. Each figure represents the best accuracy obtained by the gene-selection condition shown as the value in the parenthesis. The greatest accuracy of each data set is shown as bold letters. Values in parentheses are numbers of diagnostic genes selected by recursive feature elimination for MC-SVM, shrinkage parameters for SC, and threshold p-value for others.

	*11-SIS*	*11-SHS*	*1R-SIS*	*1R-SHS*	*1A-SIS*	*1A-SHS*	*AA-SHS*	MC-SVM	SC
thyroid	**79.8 **(10^-3^)	**79.8 **(10^-3^)	74.8 (10^-3^)	74.8 (10^-3^)	**79.8 **(10^-4^)	**79.8 **(10^-4^)	79.0 (10^-4^)	74.8 (2000)	74.8 (0.5)
GCM	86.3 (10^-3^)	86.3 (10^-3^)	89.0 (10^-5^)	89.0 (10^-5^)	89.0 (10^-6^)	89.0 (10^-6^)	**90.4 **(10^-6^)	80.8 (32)	74.0 (0)
SRBCT	100	100	100	100	100	100	100	100 (32~2308)	100 (1~2)
esophageal	72.3 (10^-1^)	72.3 (10^-1^)	71.6 (10^-1^)	71.6 (10^-1^)	73.1 (10^-1^)	**73.8 **(10^-1^)	**73.8 **(10^-1^)	71.7 (8)	68.8 (≥ 1)
