# Supplementary material for: A multi-class predictor based on a probabilistic model: application to gene expression profiling-based diagnosis of thyroid tumors
Source: BMC Genomics. 2006 Jul 27;7:190. doi: 10.1186/1471-2164-7-190 (PMC1550728; doi:10.1186/1471-2164-7-190)
Supplement: Additional File 2 — contains explanation of the data file 1. [file 1471-2164-7-190-S2.pdf]

thyroid.xls

This file contains gene expression data used in the work described in the main text.

The file contains three sheets.

Sheet "annotation": column A, in_house ID (GS and LT); column B, RefSeq ID; Column C, annotation according to RefSeq.

Sheet "learning": This file contains data used as the learning set. Column A shows in_house ID, and other columns contain gene expression data. The first row indicates tissue types. FA, follicular adenoma; FC, follicular carcinoma; PC, papillary carcinoma; N, normal thyroid tissues.

Sheet "test": This file contains data used as the test set. Column A shows in_house ID, and other columns contain gene expression data. The first row indicates tissue types.
